# Adding More Junior Residents May Worsen Emergency Department Crowding

**DOI:** 10.1371/journal.pone.0110801

**Published:** 2014-11-04

**Authors:** Takahisa Kawano, Kei Nishiyama, Hiroyuki Hayashi

**Affiliations:** 1 Department of Emergency Medicine, University of Fukui Hospital, Yoshida county, Fukui Prefecture, Japan; 2 Department of Primary Care and Emergency Medicine, Kyoto University Graduate School of Medicine, Kyoto city, Kyoto Prefecture, Japan; 3 Department of General Medicine, University of Fukui Hospital, Yoshida county, Fukui Prefecture, Japan; University of Utah School of Medicine, United States of America

## Abstract

**Background:**

Although increasing staff numbers during shifts when emergency department (ED) crowding is severe can help meet patient demand, it remains unclear how different types of added staff, particularly junior residents, may affect crowding.

**Methods:**

To identify associations between types of staff and ED crowding, we conducted a cross-sectional, single-center study in the ED of a large, teaching hospital in Japan between January and December 2012. Patients who visited the ED during the study period were enrolled. We excluded (1) patients previously scheduled to visit the ED, and (2) neonates transferred from other hospitals. During the study period, 27,970 patients were enrolled. Types of staff analyzed were junior (first and second year) residents, senior (third to fifth year) residents, attending (board-certified) physicians, and nurses. A generalized linear model was applied to length of ED stay for all patients as well as admitted and discharged patients to quantify an association with the additional staff.

**Results:**

In the model, addition of one attending physician or senior resident was associated with decreased length of ED stay for total patients by 3.88 or 1.64 minutes, respectively (95% CI, 2.20–5.56 and 0.81–2.48 minutes); while additional nursing staff had no association. Surprisingly, however, one additional junior resident was associated with prolonged length of ED stay for total patients by 0.97 minutes (95% CI 0.37–1.57 minutes) and for discharged patients by 1.01 minutes (95% CI 0.45–1.59 minutes).

**Conclusion:**

Staffing adjustments aimed at alleviating ED crowding should focus on adding more senior staff during peak-volume shifts.

## Introduction

Emergency department (ED) crowding, in which patient care cannot be provided within a reasonable time due to excess demand for emergency services, is a serious and challenging problem [1], [2]. In Japan, 13,100 patients in critical condition experienced an ambulance diversion more than four times during one ambulance transportation; about 21% of these ambulance diversions were caused by ED crowding [3]. While ED crowding decreases an ED's capacity to accept patients who are transported by ambulance service, it can also have serious consequences: the mortality of patients admitted from the ED has been shown to increase when ED crowding is severe [4], [5]. Additionally, ED crowding decreases ED physician productivity and satisfaction [6]. Importantly, a study of the association between hospital resources and ED crowding demonstrated that an increased ratio of ED staff to patient volume shortened patient treatment time in the ED [7].

When ED crowding is severe, adding more staff, e.g., board-certificated ED physicians, residents, and nurses, can be an effective countermeasure [2]. In Japan, however, hospitals commonly add only junior (first and second year) residents on shifts with high patient volumes. This practice arose from a shortage of board-certified ED physicians; the current number of these physicians is just 3,400 [8]. Meanwhile, the number of hospitals with EDs was 39,00 in 2008, and the total number of patients visited at ED in Japan was 0.57 million per day in 2011 [9], [10]. Although the additional allowance was paid to emergency physicians by government to increase number of them, the shortage is widespread in Japan. It creates staffing difficulties; adding more junior residents on shift instead of adding more board-certificated emergency physicians when ED crowding is severe. However, the contribution of junior residents to alleviating ED crowding has been questioned [11]. Thus, although the prevailing ED staffing plan in Japan might exacerbate ED crowding, this hypothesis has not yet been proven.

In this study, we used a generalized linear model to investigate the association of staff type—junior or senior resident, nurse, and attending (board-certified) physician—on the length of ED stay. The findings will provide basic information that supports the need for an increase the number of board-certificated ED physicians in Japan.

## Methods

### Study design and setting

A cross-sectional, single-center study was conducted in the ED of the Fukui Prefectural Hospital in Fukui, Japan. The Fukui Prefectural Hospital, a large teaching hospital in an urban setting, has the largest ED in Fukui Prefecture, where approximately 380,000 residents live. The area is also served by a second ED in a large academic hospital. These hospitals serve both urban and rural communities in an area of 830 km^2^. The population density of this area is 450 persons per km^2^. Seven fire departments and 7 emergency dispatch centers are located in this area. Fukui Prefectural Hospital has about 1,080 beds and 14 intensive care unit beds. The main treatment location in our ED has four beds for serious cases or patients being resuscitated. There are also four examination rooms for low-acuity patients and four beds for observation. Almost all patients who visited this ED were self-referred. The ED accepts all patients regardless of their age, including pediatric patients. In our department, nursing staff triages the new arrival patients like at other institutions. Residents are responsible for the first encounter to evaluate new arrival patients after this triage. However, nursing staff can skip this evaluation; nursing staff chooses patients with high acuity and allocates them to ED beds where senior residents and attending physicians treat and evaluate patients. All other specialist consultants are available when necessary.

### Selection of participants

Patients who visited the ED of Fukui Prefectural Hospital from 08:00 January 1, 2012 to 07:59 December 31, 2012 were enrolled in our study. We excluded (1) patients who were scheduled for admission or pharmaceutical prescription, and (2) neonates requiring intensive care who were transferred from other hospitals. The regular reception desks at our hospital are closed on weekends, therefore patients for scheduled admission or pharmaceutical prescription visit the reception desk at the ED. Patients who need pharmaceutical prescription mainly receive daily intravenous antibiotic administration at outpatient clinics. These patients quickly depart the ED without any evaluation or treatment, so the length of ED stay of these patients is quiet small. Neonates requiring intensive care transferred from another hospital typically stay in the ED during registration only, so their length of ED stay is also small. Because these patients rarely consume any ED resources beyond registration, we excluded them from this study.

### Study outcome

Study outcome was the length of ED stay, which is one of the most common proxy measures of ED overcrowding [12]. The length of ED stay was defined as the time, in minutes, from registration in ED to ED departure.

### Definitions

To assess the association of staffing types with ED crowding, we chose the number of staff (junior resident, senior resident, attending physician, and nurse) and common covariates of ED crowding in our analysis (number of walk-in patients, number of ambulance arrivals, mode of arrival, bed occupancy, ED boarding, patient age, arrival time, percentage of admission).

Junior residents were defined as first- and second-year residents. Senior residents were defined as third- to fifth-year residents. Attending physicians were defined as board-certified in Emergency Medicine and working at the ED for over six years. Junior residents determine the disposition of the patient after consulting with senior residents or attending physicians; senior residents and attending physicians determine the disposition of patients by themselves. Number of walk-ins was defined as the number of walk-in patients arriving in the ED each hour [4]. The number of ambulance arrivals was determined hourly. Mode of arrival was defined as the method of patient entry to the ED, by ambulance or walk-in [13]. Bed occupancy was defined as the percentage of hospital beds occupied per day [14]. ED boarding was defined as the number of patients at each hour who remained in the ED for more than 1 hour after admission by an ED physician [14]. Arrival time was classified into periods (08:00–15:59, 16:00–23:59, and 00:00–07:59). The percentage of admissions represented the percentage of total hospital admissions generated from the ED per day [15].

### Measurement

The number of junior and senior residents, attending physicians, and nurses was obtained from recorded work schedules. We obtained the data from the electronic medical record system (EMR) and the electronic hospital administrative data system of our hospital. Patient age, arrival time, time of decision to admit, departure time, disposition of patient, mode of arrival, and execution of any diagnostic tests were obtained from EMR. This record is considered to be accurate because every patient account is generated from it. When a patient needs to be admitted, the physician orders admission through EMR. Nurses follow the necessary procedures after this order and transfer patients to hospital wards. If patients have to wait in the ED for the arrangement of a hospital bed, nurses record these patients and provide care to them appropriately. We obtained this nursing record to calculate ED boarding. ED clerks obtained the number of available hospital beds at 09:00 each day from the electronic hospital administrative data system and calculated hospital bed occupancy. We could not obtain hourly hospital bed occupancy information.

### Statistical analysis

Descriptive analyses were conducted with the use of frequency tabulations. We described the medians and interquartile ranges (IQR) for all variables.

### Primary analysis

We defined 280 minutes, which was the upper 5% of length of ED stay for total patients when no junior resident was on shift, as a clinically significant delay. To assess the contribution of junior residents to ED crowding, we classified total patients according to the number of junior residents on shift and described the proportion of the number of patients who stayed at the ED for more than the clinically significant delay in each category. We conducted the Cochran-Armitage test to assess the presence of an association between the proportion of patients with significant delay in each category and the number of junior residents.

### Secondary analysis

To assess the association of staff types in the ED with length of ED stay, a generalized linear model (GLM) with a log-link function and gamma distribution was conducted. In this study, length of ED stay was not normally distributed (absolute skew value 2.3, absolute kurtosis 10.0) and corresponded to findings of previous studies [14], [16], [17]. GLM is commonly used to analyze length of ED stay to fit skewedness and can report the results in the original time units as opposed to a log-transformed outcome [14], [16]. The distribution of this model was determined by result of modified park test [18]. This model can determine the range of change in length of ED stay when each variable changes per its original unit and other variables are fixed to their mean.

We constructed the GLM to assess the association of each staff type with length of ED stay in the total patient group with other covariates. Next, patients were stratified into discharged and admitted groups. The model was then constructed in each group to investigate the association of each staff type with ED stay in the different acuity groups.

All analyses were conducted with STATA version 12.1 (STATA Corp LP, College Station, TX). The authors had full access to the data and take responsibility for its integrity. All authors have read and agreed to the content of the manuscript.

### Ethical statement

The study protocol was based on the guidelines for epidemiologic studies issued by the Ministry of Health, Labor and Welfare of Japan. [19] Fukui Prefectural Hospital ethics committees approved the research protocol. All data was collected retrospectively. All patient data was anonymous when researchers accessed it. Our study didn't include any personally identifiable information, so the requirement of oral or written informed consent was allowed to be waived by the ethics committees [19], [20]. So, we didn't obtain oral or written informed consent from participants. We posted the poster about our study protocol on the bulletin board at the gate of ED instead of informed consent.

## Results

### Characteristics of study participants

**Figure 1:**
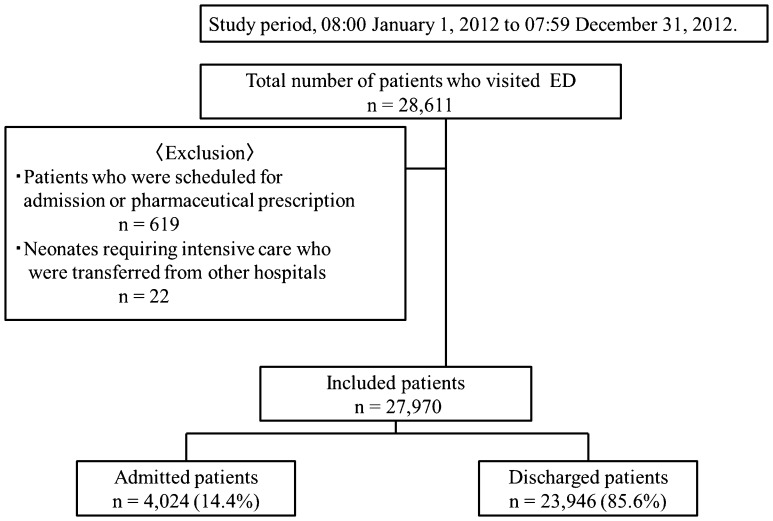
Patient recruitment diagram. ED, emergency department.

During the study period, 28,611 patients visited the ED. Of these, 619 patients who were scheduled to visit the ED for admission or pharmaceutical prescription as well as 22 newborn babies who were transferred from other hospitals were excluded. The study therefore included 27,970 (97.8%) of the total patients who visited the ED. Of the included patients, 4,024 (14.4%) were admitted to the hospital, and 23,946 (85.6%) were discharged (Figure 1).

### Patient demographics and primary outcomes

The median (quartiles) age of participants was 38 years [interquartile range (IQR), 17–74 years] (Table 1). The median daily number of ambulances was 10 cars (IQR, 8–13 cars). Of included patients, 13.5% were transported by ambulance. The median daily number of walk-in patients was 56 (IQR, 48–76). The number of walk-in patients peaked between 16:00 to 23:59 (37: IQR, 31–44). The median length of ED stay for total patients was 88 minutes (IQR, 53–147 minutes). The median length of ED stay for admitted and discharged patients were 170 and 70 minutes, respectively (IQR, 117–233, and 50–128 minutes).

**Table 1:** Patient demographics and primary outcomes.

	n = 27,970
Median age, years (IQR)	38 (17–64)
Walk-in patients, n	24,186
per day, n (IQR)	56 (48–76)
08:00 to 15:59, n (IQR)	19 (15–34)
16:00 to 23:59, n (IQR)	37 (31–44)
0:00 to 07:59, n (IQR)	11 (8–14)
Ambulance, n	3784
per day, n (IQR)	10 (8–13)
08:00 to 15:59, n (IQR)	6 (3–6)
16:00 to 23:59, n (IQR)	4 (3–6)
0:00 to 07:59, n (IQR)	2 (1–3)
Admissions, n	7,843
per day, n (IQR)	11 (8.3–13)
Percentage of admission, % (IQR)	13.7 (9.8–18.0)
Hospital occupancy	
per day, % (IQR)	68.5 (66.3–70.7)
ED boarding	
per day, n (IQR)	2 (1–3)
Deaths, n (%)	96 (0.3)
Median Length of ED stay	
Total patients, min (IQR)	88 (53–147)
Admitted patients, min (IQR)	170 (117–233)
Discharged patients, min (IQR)	70 (50–128)

IQR, interquartile range; ED, emergency department.

### Staffing in the ED

The median hourly numbers of junior residents, senior residents, attending physicians, and nurses on shift were four, two, one, and three, respectively (IQR: 2–5, 1–3, 1–2, and 2–3)(Table 2). The median hourly number of junior residents, senior residents, and nurses peaked between 16:00 to 23:59 (4: IQR, 4–5; 2: IQR, 1–3; and 3: IQR, 3–3; respectively). This peak in the number of staff was consistent with the peak in number of walk-in patients.

**Table 2:** Staffing in ED.

	Median (IQR)
Junior resident	
per hour, n	4 (2–5)
08:00 to 15:59, n	2 (1–4)
16:00 to 23:59, n	4 (4–5)
0:00 to 07:59, n	3 (3–4)
Senior resident	
per hour, n	2 (1–3)
08:00 to 15:59, n	1 (1–2)
16:00 to 23:59, n	2 (1–3)
0:00 to 07:59, n	1 (1–1)
Attending physician	
per hour, n	1 (1–2)
08:00 to 15:59, n	1 (1–2)
16:00 to 23:59, n	1 (1–2)
0:00 to 07:59, n	1 (1–1)
Nurse	
per hour, n	3 (2–3)
08:00 to 15:59, n	2 (2–2)
16:00 to 23:59, n	3 (3–3)
0:00 to 07:59, n	2 (2–2)

IQR, interquartile range; ED, emergency department.

### Primary analysis

**Figure 2:**
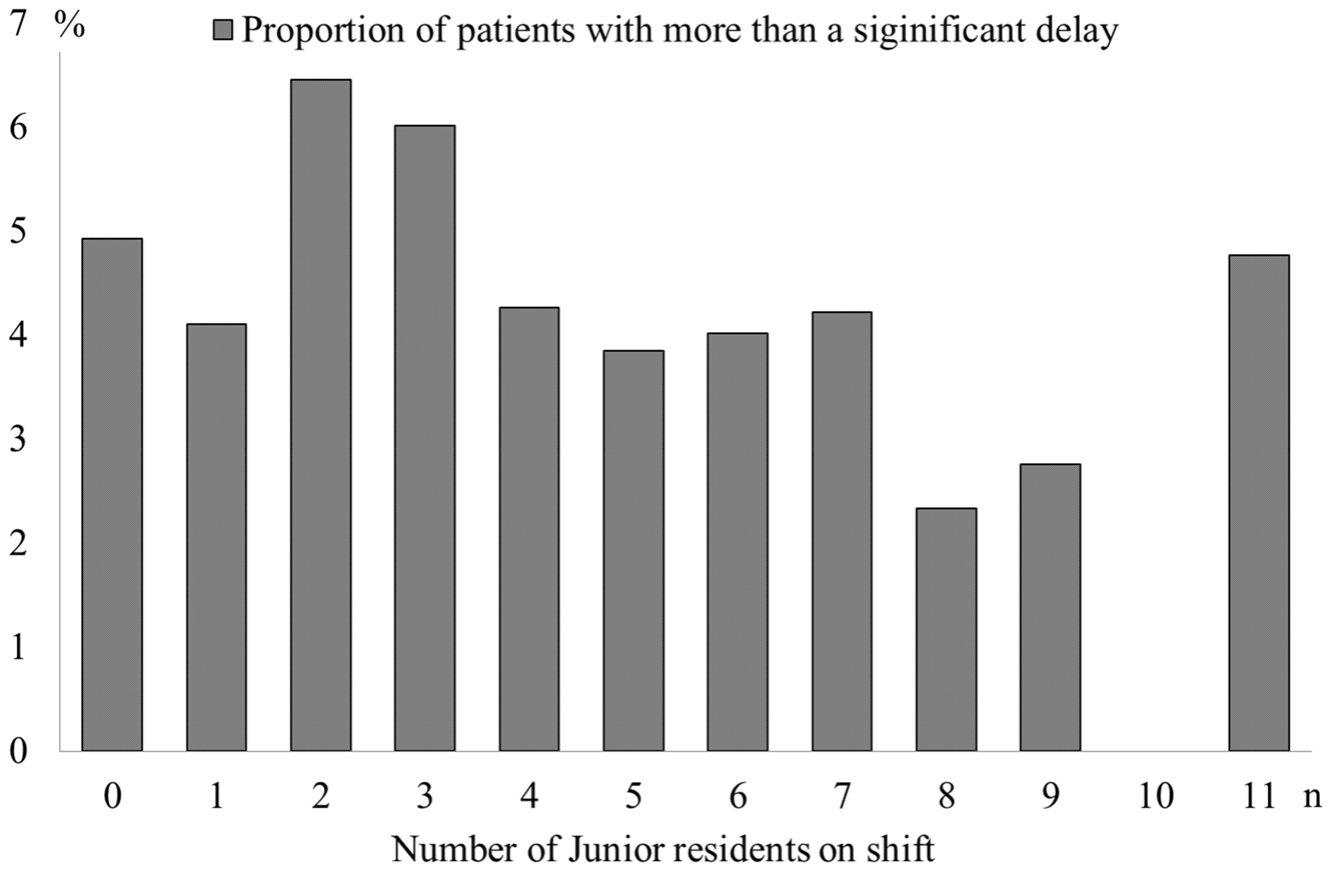
Proportion of patients with a significant delay in each category classified by the number of junior residents on shift.

The proportion of patients with a clinically significant delay is described in Figure 2. A statistically significant association between the proportion of patients with a significant delay in each category and the number of junior residents was not detected (P = 0.054).

### Secondary analysis

The GLM was applied to estimate the associations of changes in ED staffing on length of stay in ED. In our model, adding one attending physician was associated with decreased length of ED stay for total patients (3.9 minutes, 95% CI, 2.2–5.5 minutes)(Table 3). Adding one senior resident was associated with decreased length of ED stay (1.6 minutes, 95% CI, 0.8–2.5 minutes). However, the range of decrease in length of ED stay when adding a senior resident was smaller than for an attending physician (−1.6 vs. −3.9 minutes). In contrast, adding one junior resident was associated with prolonged length of ED stay for total patients (1.0 minutes, 95% CI, 0.4–1.6). Adding one nurse did not have a statistical association on length of ED stay for total patients.

**Table 3:** The range of change in length of ED stay for total patients when each variable changed per its original unit.

	Total patients (n = 27,970)	
Characteristic	Length of stay, minutes (Unstandardized slope coefficient)	95% CI
Mode of arrival		
Walk-in	Reference	
Ambulance	30.4*	27.1–33.6
Number of walk-in patients	0.4	-<0.1–0.7
Number of ambulance arrivals	3.6*	2.3–4.9
Staff in ED		
Junior resident	1.0*	0.4–1.6
Senior resident	−1.6*	−2.5–−0.8
Attending physician	−3.9*	−5.6–−2.2
Nurse	−0.6	−2.4–1.2
Bed occupancy	0.4*	0.7–0.7
ED boarding	−0.4	−3.3–1.3
Patient age	0.8*	0.8–0.9
Arrival		
08:00–15:59	Reference	
16:00–23:59	−4.2*	−7.0–−1.4
0:00–07:59	2.5	−0.9–5.9
Percentage of admissions	0.9*	0.8–1.1

*, P<0.05; 95% CI, 95% confidence intervals; ED, emergency department

The model was then applied to patients grouped by admitted and discharged status. Adding one attending physician was associated with decreased length of ED stay for both admitted (−6.5 minutes, 95% CI, −1.2–−11.8 minutes) and discharged patients (−4.0 minutes, 95% CI, −2.4–−5.6 minutes)(Table 4). The range of change in length of ED stay was wider in admitted patients than in discharged patients. Similarly, adding one senior resident was associated with decreased length of ED stay for discharged patients (−1.9 minutes, 95% CI, −1.1–−2.7 minutes). However, adding a senior resident did not have a statistical association with the length of ED stay for admitted patients. Interestingly, adding one junior resident on shift was associated with prolonged length of ED stay for discharged patients (1.0 minutes, 95% CI 0.5–1.6 minutes) but had no statistical association with length of ED stay for admitted patients. An additional nurse had no statistical association with length of ED stay for either patient group.

**Table 4:** The range of change in length of ED stay for admitted and discharged patients when each variable changed per its original unit.

	Admitted patients (n = 4,024)		Discharged patients (n = 23,946)	
Characteristic	Length of stay, minutes (Unstandardized slope coefficient)	95% CI	Length of stay, minutes (Unstandardized slope coefficient)	95% CI
Mode of arrival				
Walk-in	Reference		Reference	
Ambulance	−41.6*	−49.4–−33.8	39.3*	25.7–42.9
Number of walk-in patients	1.6*	0.3–2.9	0.4*	0.6–0.8
Number of ambulance arrivals	0.3	−4.0–4.6	3.4*	2.2–4.7
Staff in ED				
Junior resident	0.4	−1.5–2.4	1.0*	0.5–1.6
Senior resident	−1.0	−3.7–1.7	−1.9*	−2.7–−1.1
Attending physician	−6.5*	−11.8–−1.2	−4.0*	−5.6–−2.4
Nurse	0.4	−6.0–6.9	0.2	−1.5–2.0
Bed occupancy	0.1	−0.9–1.1	0.6*	0.3–0.9
ED boarding	6.1	−1.4–13.7	−0.9	−3.1–1.3
Patient age	0.5*	0.4–0.6	0.7*	0.7–0.7
Arrival				
08:00–15:59	Reference		Reference	
16:00–23:59	−13.6*	−22.8–−4.4	−1.8	−4.4–0.9
0:00–07:59	37.2*	26.3–48.0	2.3	−0.9–5.5
Percentage of admissions	0.4	−0.1–1.0	0.4*	0.3–0.6

*, P<0.05; 95% CI, 95% confidence intervals; ED, emergency department.

## Discussion

Using actual ED data to supply our model, we demonstrated the contribution of each type of ED staff to the length of ED stay. Our model is consistent with previous studies, in that the addition of an attending physician was associated with reduced length of ED stay for both admitted (by ∼6.5 minutes) and discharged (by ∼4 minutes) patients [11]. The addition of one senior resident in the model was associated with reduced length of ED stay, but only for discharged patients (by ∼2 minutes). Interestingly, in contrast, an additional junior resident was associated with prolonged length of ED stay for total patients and discharged patients (by ∼1.0 minute each). The addition of one nurse had no statistical association with the length of ED stay for any patient group. These findings indicate that the addition of senior physicians can improve ED crowding, but the addition of junior physicians might worsen ED crowding.

To provide quality care within an appropriate timeframe when ED crowding is severe, adding staff helps meet patient volume [2]. However, the contribution of residents to the alleviation of ED crowding has been debated. For example, during a resident strike in Australia, ED shifts were comprised of board-certified physicians, and the total number of physicians on shift was lower than before the strike; however, the length of ED stay was lower during the strike [11]. Our findings support and extend this previous study; we estimated the associations of each type of medical staff with length of ED stay directly. The results suggest that, when demand is estimated to be high in the ED, staffing managers should consider adding senior residents or attending physicians to shifts rather than junior residents.

Our study emphasizes the role of board-certificated ED physicians in alleviating ED crowding; only this type of staff was associated with reduced length of ED stay of both admitted and discharged patients. To improve ED efficacy, hospital administrators and Japanese government officials should increase efforts to end the shortage of ED physicians. In Japan, medical students have a right of free selection of their specialties, and their selections are influenced by the advice of senior doctors [21]. However, 32% of senior residents who studied emergency medicine were reportedly not satisfied with their specialty [22]. In particular, the stress of working in the ED was the primary factor contributing to their dissatisfaction compared to other factors including their working conditions, and training and teaching systems at ED [22]. To increase the number of board-certificated ED physicians, measures should focus on relieving stressful conditions for ED residents, thereby encouraging medical students to select Emergency Medicine as their specialty. Such measures will be increasingly important, as ED crowding in Japan is estimated to become more severe due to the advancing age of the population [23].

Patient volume in the ED is considered to be relatively predictable [2]. The National Hospital Ambulatory Medical Care Survey indicated that patient volume in EDs in the U.S. is highest in the evening and on Mondays, Tuesdays, and Sundays than on other days of the week [16]. Additionally, the percentage of admissions from the ED on weekdays was higher than that on weekends [24]. Thus, ED crowding is likely severe on Monday and Tuesday evenings, with a high percentage of admissions from ED, and on Sundays, with a low percentage of admissions. From our findings, one could propose that adding another attending physician to the usual shift staff on Monday and Tuesday evenings would be the best action, as attending physicians can reduce length of ED stay for admitted patients. When ED crowding is severe on Sundays, adding an attending physician or senior resident could reduce the length of stay (for discharged patients only, in the case of senior residents). Further, analysis of patient arrival patterns and admission rates for particular times could allow even more effective shift scheduling to meet patient demand.

In this study, the peak in number of staff on shift was consistent with the peak in number of walk-in patients. However, the additional staff on shift comprised junior and senior residents and nurses. According to our model, this staffing plan in our hospital may be, in part, responsible for exacerbated ED crowding. The addition of five junior residents on a shift when ED crowding is severe would add another five minutes to the length of ED stay for each patient during the shift; thus, the additional residents will not alleviate ED crowding. This approach to ED staffing is common in Japan; however, our study demonstrates that this prevalent staffing plan is not successful for reducing ED crowding.

Two phenomena may explain why the addition of a junior resident contributed to increased length of ED stay. First, junior residents likely require more time to evaluate patients. One study found that when an ED patient was initially assessed by an attending physician, compared to a resident, the length of ED stay was shorter. [1]. Second, teaching hospitals typically require that a senior resident or attending physician evaluates and treats a patient after initial evaluation by a junior resident. This additional step introduces a delay that may prolong ED stay [25]. However, high-acuity patients are typically not evaluated by a junior resident first, as attending physicians or senior residents will begin evaluation and treatment immediately. In our study, the addition of a junior resident was associated with a prolonged length of ED stay only for discharged patients. Thus, the time required for resident evaluation and follow-up by senior physicians in lower-acuity patients may have led to the lengthened stay identified in our study.

Patient volume in this study might have a smaller association with length of ED stay than it would in large EDs. However, large EDs having annual patients volumes of over 60,000 are less common, accounting for only 11% of total EDs in U.S. [26]. Although our ED volume is smaller, matching the annual ED volume of 20,000–40,000 in mid-sized U.S. EDs, these smaller EDs are more common, accounting for 25% of total EDs in the U.S. [26]. The trend of increasing associations of input factors on ED stay when patient volume is higher is observed even at smaller EDs with annual patient volumes of 20,000 [26]. Thus, our findings may be beneficial for mid- and smaller-sized EDs.

### Limitations

Several limitations existed in our study. First, this study is a model-based analysis; our findings need to be validated in experimental situations. Second, the data were obtained from a single institution over just one year in Japan, which might reduce the generalizability of the conclusions. Our approach corresponds to that of previous studies of ED crowding in a single site [14]. Our findings may have limited generalizability due to the differences in health care systems between Japan and other nations, but are considered to be generalizable within Japan, especially for other teaching hospitals. Third, although ambulance diversion and patients leaving before evaluation are common proxy measures of ED crowding, we did not include them in this study because we did not measure them. Fourth, we could not obtain triage results, so patients were not classified according to level of disease severity. Patients who were considered as high acuity at triage but were eventually discharged from the ED might have a different time of assessment by senior residents because of difficulty of case management. Additionally, the range of decrease in length of ED stay of discharged patients by senior residents may be shortened due to these complex patients. Fifth, a lack of hourly bed occupancy counts in our data system precluded analysis of hourly hospital bed occupancy. Since bed occupancy changes each hour, an ideal analysis would be performed according to the precise hospital bed occupancy at a particular time. Despite this limitation, our findings are supported by previous studies that found an association between length of ED stay and daily bed occupancy [15], [27]. Finally, we cannot exclude possible bias from unmeasured and unavailable factors such as the time involved in awaiting laboratory or radiology results and consultation from other specialists.

## Conclusions

Our statistical model indicates that different types of ED staff make different contributions to length of patient stay in the ED. In particular, the addition of attending physicians and senior residents is associated with reduced ED stays. In contrast, adding a junior resident may prolong ED stays, which may worsen ED crowding. To improve efficacy at EDs in Japan, government and health care authorities should place on emphasis on addressing the shortage of board-certificated ED physicians.

## Supporting Information

Supporting Information S1 **Database.**
(XLSX)
